# Quercetin of huoxuehuayu tongluo decoction and azithromycin combination therapy effectively improves rat tubal factor infertility by inhibiting inflammation

**DOI:** 10.22038/IJBMS.2024.72049.15662

**Published:** 2024

**Authors:** Liang Shao, Nansu Wang, Yan Yan, Yali Tan, Qin Wu, Lei Lei, Mingming Wang, Ling Liu

**Affiliations:** 1 Department of Emergency, the First Affiliated Hospital of Hunan Traditional Chinese Medicine College, Zhuzhou, Hunan, China; 2 Department of TCM Gynaecology, the First Affiliated Hospital of Hunan Traditional Chinese Medicine College, Zhuzhou, Hunan, China; 3 Combine traditional Chinese and Western Medicine Institute, Hunan University of Chinese Medicine, Changsha, Hunan, China; 4 Clinical Laboratory, the First Affiliated Hospital of Hunan Traditional Chinese Medicine College, Zhuzhou, Hunan, China

**Keywords:** Azithromycin, Inflammation, Pregnancy, Quercetin, Tubal factor infertility

## Abstract

**Objective(s):**

Tubal factor infertility (TFI) is common female infertility responsible for a large portion of female factor infertility. This study reveals the effect of the quercetin of Huoxuehuayu Tongluo Decoction with azithromycin on the pregnancy rate and inflammation of TFI female rats.

**Materials and Methods:**

Female Sprague Dawley rats were constructed into the TFI model and treated with quercetin, Huoxuehuayu Tongluo Decoction, and combination therapy (quercetin and azithromycin). Pregnancy rate and litter size were measured. Network pharmacology was applied to analyze the interaction between Huoxuehuayu Tongluo Decoction and TFI. The combination of quercetin and IL-6 was analyzed by molecular docking. HE staining and electron microscopy were used to observe the histopathology and ultrastructure of fallopian tube tissues. The TNF-α, IL-1β, IL-6, IL-8, and MPO levels were detected by ELISA. The activation of JAK/STAT, MAPK, and NF-κB p65 pathways was detected by western blot or immunohistochemistry.

**Results:**

Quercetin was the main active component of Huoxuehuayu Tongluo Decoction, and could bind to IL-6 in TFI. Target genes were enriched in the IL-17 signaling pathway, JAK-STAT signaling pathway, inflammatory disease, etc. Under the quercetin and azithromycin combination therapy, both rat pregnancy rates and litter sizes increased significantly. quercetin and azithromycin alleviated the symptoms of hydrosalpinx and inflammatory damage in fallopian tube tissues. The phosphorylation of JAK/STAT and MAPK pathways and NF-κB p65 translocation to the nucleus were significantly inhibited by the quercetin and azithromycin therapy.

**Conclusion:**

Quercetin and azithromycin combination therapy inhibited inflammation and phosphorylation of JAK/STAT and MAPK pathways to improve TFI inflammation and pregnancy function.

## Introduction

Tubal factor infertility (TFI) can be caused by multiple factors, including diseases, obstructions, structural damage, etc. (1). TFI, characterized by damaged and blocked fallopian tubes, occurs when the fallopian tubes are blocked. It may further impede a fertilized or unfertilized ovum into the uterus through fallopian tubes and prevent sperm from reaching the egg for fertilization (2). Therefore, TFI remarkably accounts for infertility and abnormal pregnancy (3). Traditionally, the treatment of TFI largely relies on antibiotic therapies based on infectious agents, such as *Chlamydia trachomatis* infection (4). The commonly used antibiotics include azithromycin, cefoxitin, or cefotetan plus doxycycline, clindamycin plus gentamicin, and ampicillin/sulbactam plus doxycycline (5). Antibiotic therapy works early on, but later, patients develop resistance and have many side effects. Traditional Chinese medicine (TCM) has been widely used as a preventive or therapeutic strategy in modern medicine (6). Therefore, we tried to relieve bacterial infection-induced TFI with TCM decoction or TCM decoction with antibiotics.

Bacterial infection, especially *Chlamydia* infection, is the primary cause of TFI (7). *Chlamydia* infection is estimated to lead to pelvic inflammatory diseases (PID) in about 35 percent of cases in women between 16 and 24 years old and about 20 percent in women aged 16 to 44 years old (8). Previous researchers have demonstrated the tight connection between signaling-induced inflammation and TFI, and this signaling includes pathways such as the JAK/STAT pathway (9), the NF-κB pathway (10, 11), and the ERK/JNK/p38 pathway (12, 13). Inflammation allows fallopian tubes to exhibit multiple histomorphological changes, including swelling, hydrosalpinx, tissue adhesion, mucosal congestion, exudation, and lumen stenosis or even occlusion (7, 14). Through the phosphorylation of critical effector proteins in the pathways to fulfill downstream regulations, all these well-established signaling pathways considerably contribute to releasing a wide spectrum of pro-inflammatory cytokines, which further triggers inflammation (15). Therefore, improving inflammation related to bacterial infection may be an effective method to treat TFI.

Two traditional Chinese herbal decoctions, Huoxuehuayu Decoction and Tongluo Decoction have the functions of promoting blood circulation and dredging collateral, respectively (16, 17). Huoxuehuayu Decoction was widely used as a therapeutic regimen for diseases that were accompanied by blood stasis (18). Its function in improving blood circulation has been well-approved in different research (18-20). Being effective in dredging collateral, Tongluo Decoction has been used in modern biomedical studies and therapies (21, 22). For example, it played a role in blocking the apoptotic mitochondrial pathway and inhibiting cardiomyocyte apoptosis (23). Inflammation was one of the prevailing reasons that contributed to TFI formation. There were accumulating reports about the therapeutic effects of Huoxuehuayu Decoction and Tongluo Decoction on inflammation (18, 24, 25). Huoxuehuayu Tongluo Decoction was combined with Huoxuehuayu Decoction and Tongluo Decoction. It consists of Angelica, *Rehmannia glutinosa*, peach kernel, safflower, Citrus Aurantium, red peon, *Ligusticum chuanxiong hort*, bupleurum, *Platycodon grandiflorum*, Achyranthes, licorice, pangolin, Lulutong, loofah, Saponaria thorns, *Melia toosendan Sieb. et Zucc*, and *Caulis spatholobi*. As a result, we speculated that the therapy of Huoxuehuayu Tongluo Decoction serves as a potential cure for treating bacterial-induced TFI by improving inflammation. 

At present, interventional recanalization combined with TCM can significantly ameliorate the pathological condition of the fallopian tube after treatment (26). Combined chitosan and danshen injections were effective in preventing fallopian tube obstruction after interventional recanalization and may increase pregnancy rates in infertile women (27, 28). Azithromycin was widely used in the antibiotic treatment of reproductive tract infections and sexually transmitted infections and has fewer clinical failures or adverse events (29, 30). Here, we attempt to analyze the potential mechanism of action of Huoxuehuayu Tongluo Decoction through network pharmacology and explore the efficacy of Huoxuehuayu Tongluo Decoction and its active ingredients combined with azithromycin in the treatment of bacterial infection-induced TFI rats. Our study provided new insight to expand the use of TCM in infertility.

## Materials and Methods

***Treatment of rats with bacteria***

A total of SPF 100 female rats (250~300 g, 8~10 weeks), which were sexually mature but not mated were used and purchased from Hunan Slike Jingda Laboratory Animal Co., Ltd. A group of 20 Sprague Dawley (SD) female rats was used as the Control without further treatment. The rest were treated with a mixture of multiple bacteria to induce TFI rats, including *Escherichia coli*, *Staphylococcus aureus*, and *Streptococcus agalactiae*, diluted with sterile saline at a ratio of 2:1:1 (1). After the treatment, three rats of each group were randomly selected on day 28 to observe the histomorphological changes of the fallopian tubes and the occurrence of edema, swelling, hydrosalpinx, and adhesions of surrounding tissues. After cutting the lumen, a successful model featured the symptoms of mucosal congestion, exudation, adhesions, water accumulation, and lumen stenosis or even occlusion. Based on the severity of hydrosalpinx, five levels (Lv. 0-4) were used to assess modeled fallopian tubes: no hydrosalpinx (Lv. 0), detected hydrosalpinx only after expansion (Lv. 1), visible hydrosalpinx to the naked eye with a size smaller than (Lv. 2), equal to (Lv. 3), and larger than (Lv. 4) that of the ipsilateral ovary.

***Preparation of Huoxuehuayu Tongluo Decoction***

The compound of Huoxuehuayu Tongluo Decoction was supplied by The First Affiliated Hospital of Hunan Traditional Chinese Medicine College. Huoxuehuayu Tongluo Decoction was combined with Huoxuehuayu Decoction and Tongluo Decoction. It consists of 15 g of Angelica, 15 g of *Rehmannia glutinosa,* 15 g of peach kernel, 10 g of safflower, 10 g of Citrus Aurantium, 10 g of red peon, 10 g of *Ligusticum chuanxiong hort*, 10 g of bupleurum, 15 g of *Platycodon grandiflorum*, 10 g of Achyranthes, 5 g of licorice, 9 g of pangolin, 15 g of Lulutong, 10 g of loofah, 10 g of Saponaria thorns, 10 g of Melia *toosendan Sieb. et Zucc*, and 10 g of *Caulis spatholobi*. The specific preparation method of Huoxuehuayu Tongluo Decoction was as follows: Five doses of each of the above-mentioned Chinese medicines were prepared by the Pharmaceutical Preparation Room from The First Affiliated Hospital of Hunan Traditional Chinese Medicine College. The concentrated solution was prepared by water extraction and alcohol precipitation, and adjusted to 2 g/ml containing raw medicinal materials. The pH of the concentrated solution was adjusted to 6.5~7.0. The solution was stored at 4 ^°^C and used in the experiments.

***Treatment of rats with drug therapies***

A total of 100 female rats, including 20 controls (Group 1) and 80 modeled rats (Group 2-5), were divided into 5 groups: Control, Model, Quercetin, Huoxuehuayu Tongluo Decoction (HXHYTLD), and combination therapy (Quercetin and Azithromycin, Que+Azi) groups. Rats in the Control and Model groups had no drug intervention but received intragastric administration of distilled water. Rats in the Quercetin group were treated with quercetin (80 mg/kg/day, Q4951, Sigma-Aldrich)(31). Based on the clinical dose of Huoxuehuayu Decoction (32) and Huoxue Tongluo Decoction (33), rats in Huoxuehuayu Tongluo Decoction group were treated with Huoxuehuayu Tongluo Decoction drugs (14.4 g/kg), which was calculated according to the body surface area method (34). Rats in the Que+Azi group were gavaged with quercetin (80 mg/kg/day, Q4951, Sigma-Aldrich) and 0.4 ml/d azithromycin (6.5 g/ml, 101012, Sanchine). Intragastric administration was carried out at the same time in the morning. For examination, 10 female rats of each group were randomly selected 28 days after intragastric administration. A total of 25 male rats (250~300 g, 8~10 weeks) were used and purchased from Hunan Slike Jingda Laboratory Animal Co., Ltd. The remaining 10 female rats of each group were mated with 5 male rats in a ratio of 2:1. The pregnancy rates and average litter sizes of female rats were tracked and recorded.

***Network pharmacology analysis***

The compounds and targets of Huoxuehuayu Tongluo Decoction (Angelica, *Rehmannia glutinosa,* peach kernel, safflower, *Citrus aurantium*, red peon, *Ligusticum chuanxiong* *hort*, bupleurum, *Platycodon grandiflorum*, Achyranthes, licorice, pangolin, Lulutong, loofah, Saponaria thorns*, Melia toosendan Sieb. et Zucc*, and *Caulis spatholobi*) were searched in the TCMSP database (35) (https://tcmspw.com/tcmsp.php), and the screening conditions were OB≥30% and DL≥0.18. The GeneCards, NCBI, OMIM, and DisGeNET databases were searched using “Tubal factor infertility “ as a keyword. The screened Huoxuehuayu Tongluo Decoction targets and TFI targets were input into Venny 2.1 to obtain the common targets. The common targets of Huoxuehuayu Tongluo Decoction and TFI were input into the String database (36) (https://string-db.org/cgi/input.pl) to construct a PPI network. PPI networks were imported into Cystoscape 3.8.0 (37) for topology analysis and degree sorting by using NetworkAnalyzer tools. Based on the included components, therapeutic diseases, and targets, the Huoxuehuayu Tongluo Decoction composition-TFI-target network diagram was constructed. The composition-disease-target network diagram was imported into Cytoscape 3.8.0 for topology analysis and degree ranking. KEGG pathway enrichment analysis was performed on the common targets of Huoxuehuayu Tongluo Decoction and TFI. The items with corrected *P*-value<0.05 were screened by using the String database. The R (4.0.3) was installed and referenced to the clusterProfiler package for bubble plotting.

***Molecular docking of quercetin with IL-6 protein***

In recent years, molecular docking has played an important role in obtaining new drug candidates in a short time and at low cost through computational tools (38). Docking aims to take a close look at ligands bound to receptor-binding pockets and predict the interactions and energies between receptors and ligands. In this study, VINA 1.1.2 software was used to study the docking between quercetin and IL-6 protein, and the docking binding energy standard was less than -4.25 kcal/mol. Subsequently, Discovery Studio was used for visual analysis of quercetin and IL-6 protein.

***HE Staining***

Tissues of the fallopian tubes of 10 rats in each group were isolated and prepared for HE staining. The tissues were embedded and sectioned. The sections were dewaxed and stained with hematoxylin (Abiowell) and eosin (Abiowell), respectively. The sections were then dehydrated with gradient alcohol (95-100%) and sealed. The sections were examined on a microscope (BA210T, Motic).

***Observation of the ultrastructure of fallopian tubes under electron microscopy***

For electron microscopy, isolated tissues were soaked in 2% glutaraldehyde at 4 ^°^C for 2 hr followed by incubation with additional post-fixation in 1% OsO4 (Merck, Darmstadt, Germany) for 2 hr. After fixation, the tissues were dehydrated in a graded ethanol series and embedded in epoxy resin. Ultrathin sections were cut with an ultramicrotome and stained with saturated uranyl acetate and lead citrate. Sections were assessed at 80 kV using a Philips CM-120 transmission electron microscope.

***ELISA***

The ELISA kits for IL-1β, IL-8, IL-6, and TNF-α were purchased from CUSABIO, Wuhan, China. The kit for MPO was purchased from Nanjing Jiancheng Bioengineering Institute, Nanjing, China. The kits of IL-1β (Catalog: CSB-E08055r), IL-18 (Catalog: ml037351), TNF-α (Catalog: CSB-E11987r), IL-6 (Catalog: CSB-E04640r), and MPO (Catalog: A044-1-1) were applied to assess individual cytokine levels in fallopian tissue supernatant according to the manufacturer’s instructions, respectively. In brief, the 96-well plates were coated with antibodies against inflammatory cytokines and supernatants followed by washing with sample buffer three times. The secondary antibodies were added, followed by further incubation and wash for 5 times. TMS substrate and stop solution were sequentially added. The optical density was measured at 450 nm using a microplate reader (MB-530, huisong).

***Western blot***

Total protein samples from rat tissues were harvested and extracted using RIPA lysis buffer (AWB0136, Abiowell, China). The protein samples were prepared using a loading buffer and separated by 10% SDS-PAGE. The proteins were electrotransferred onto nitrocellulose membranes and subsequently blocked with 5% non-fat milk (AWB0004, abiowell, China) dissolved in PBS for 2 hr at room temperature. Next, the membranes were incubated with primary antibodies under specific conditions at 4 ^°^C overnight (Table 1). Membranes were incubated with the secondary antibodies (Table 1). GAPDH was used as an internal control. SuperECL Plus (AWB0005, Abiowell, China) and Quantity One software were used for visualization and imaging analysis.

***Immunohistochemistry (IHC)***

Fallopian tube tissue sections of each experimental group were used for IHC. IHC staining of p65 and IκBα protein expression was performed following the standard protocol by using the anti-p65 and anti-IκBα antibodies (Cell signaling; 1:100 dilution), followed by incubation with the secondary antibody (D-3004; Shanghai Long Island Biotec. Co., Ltd). Immunohistochemical evaluation was performed based on the percentage of positively stained cells and the staining intensity, ranging from 0 to 3.

***Statistical analysis***

All statistical analyses were carried out using GraphPad Prism 8.0.2 (GraphPad Software, San Diego, CA, USA). Data were expressed as mean±standard deviation (SD). The test conforms to the normal distribution and the variance is homogeneous. The unpaired t-test is used between groups. The comparison between multiple groups uses one-way ANOVA or ANOVA of repeated measurement data. Tukey’s *post-hoc* test is used for conducting multiple comparisons after an ANOVA. *P*<0.05 means the difference is statistically significant.

## Results

***Network pharmacology analysis of Huoxuehuayu Tongluo Decoction drug and TFI disease interaction targets***

**Figure 1:**
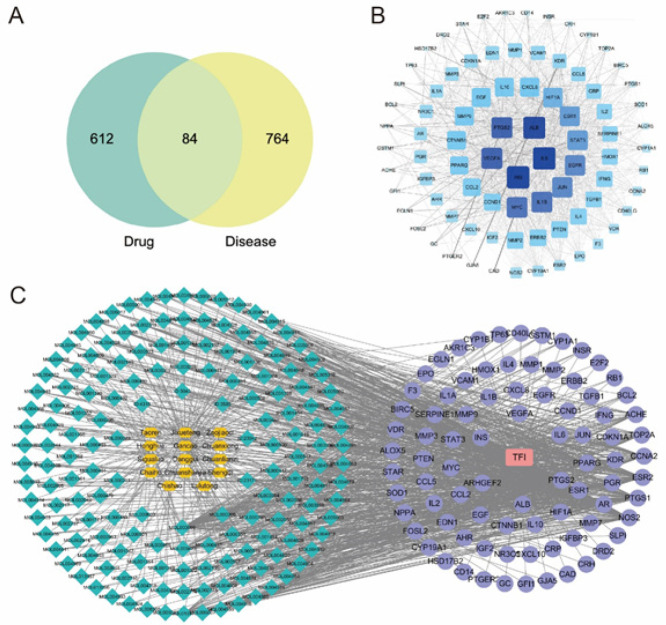
Network pharmacology analysis of Huoxuehuayu Tongluo Decoction drug and TFI disease interaction targets

Venn diagram showed that Huoxuehuayu Tongluo Decoction and TFI shared 84 targets (Figure 1A). PPI network analysis showed that IL-6, INS, ALB, PTGS2, and VEGFA were the key gene targets (Figure 1B). The component-disease target network further explained the potential interaction mechanism between Huoxuehuayu Tongluo Decoction drug components and TFI disease targets (Figure 1C). These results suggested that Huoxuehuayu Tongluo Decoction drug had gene target interaction with TFI disease.

***Degree and function of Huoxuehuayu Tongluo Decoction drug components and TFI disease target genes***

**Figure 2:**
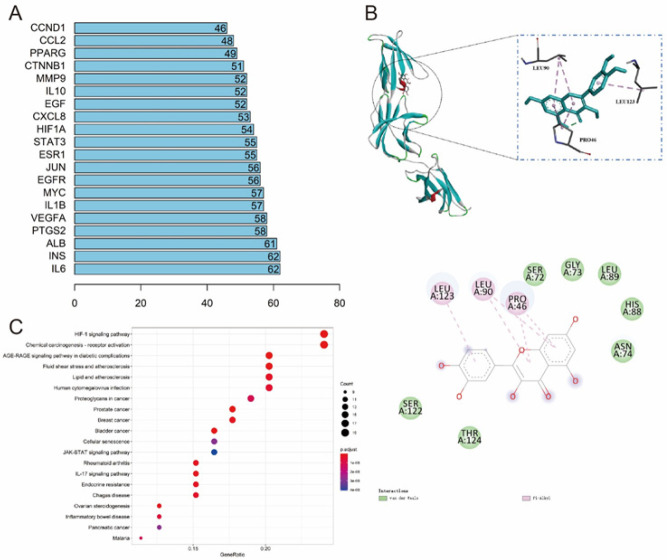
Degree and function of the target genes in Huoxuehuayu Tongluo Decoction and tubal factor infertility (TFI) disease

Key component-target topology analysis showed that quercetin (MOL000098) was the drug component with the highest degree in Huoxuehuayu Tongluo Decoction (Table 2). IL-6 and INS were the target genes with the highest degree in TFI diseases (Figure 2A). Molecular docking showed quercetin formed hydrophobic interactions with LEU90, LEU123, and PRO46 of the IL-6 protein, which were the main forces that drove the compound to bind to the active site (Figure 2B). KEGG function prediction analysis showed that the functions of Huoxuehuayu Tongluo Decoction and TFI target genes may be related to IL-17 signaling pathway, JAK-STAT signaling pathway, inflammatory bowel disease, etc. (Figure 2C). These results proved that quercetin, as the key component of Huoxuehuayu Tongluo Decoction, may improve TFI through IL-6.

***Quercetin and azithromycin combination therapy significantly improves pregnancy rates***

To examine whether the therapy effectively improved the infertility of TFI rats, we determined the pregnancy rates and the average litter sizes in different groups (Table 3). Control had the highest pregnancy rate with an average litter size of 12.6±1.51 (Table 3). By contrast, the pregnancy rate in the Model group was much lower than that in the Control group, only 40% of the Control group, evidenced by the small average litter size (Table 3). The application of either quercetin or Huoxuehuayu Tongluo Decoction to the Model strikingly improved the pregnancy rate from 40% to 70% and increased the average litter size (Table 3). Together, the quercetin and azithromycin therapy generated a higher recovery of the pregnancy rate (80%) and average litter size (10.75±1.49)(Table 3). As a result, the quercetin and azithromycin therapy has shown potency to improve the pregnancy rate in TFI rats. 

***Quercetin and azithromycin combination therapy prevents hydrosalpinx development in the fallopian tube***

**Figure 3:**
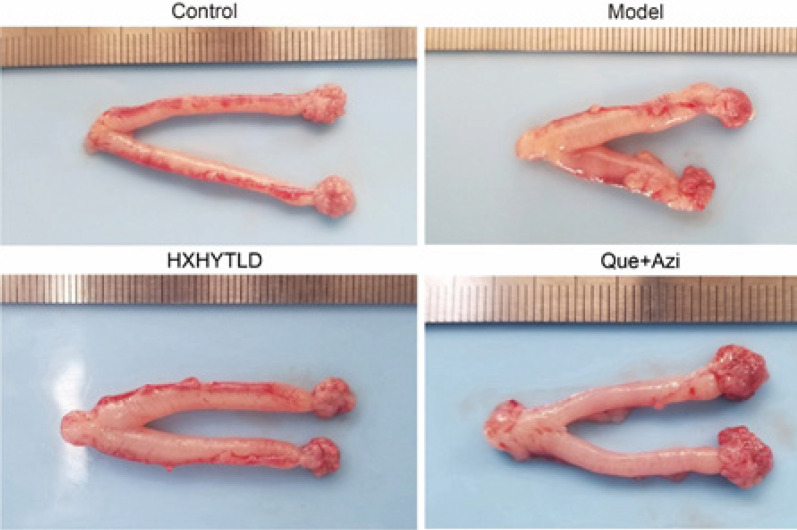
Quercetin and azithromycin combination therapy improved the Generation of hydrosalpinx in tubal factor infertility (TFI) rats

**Figure 4:**
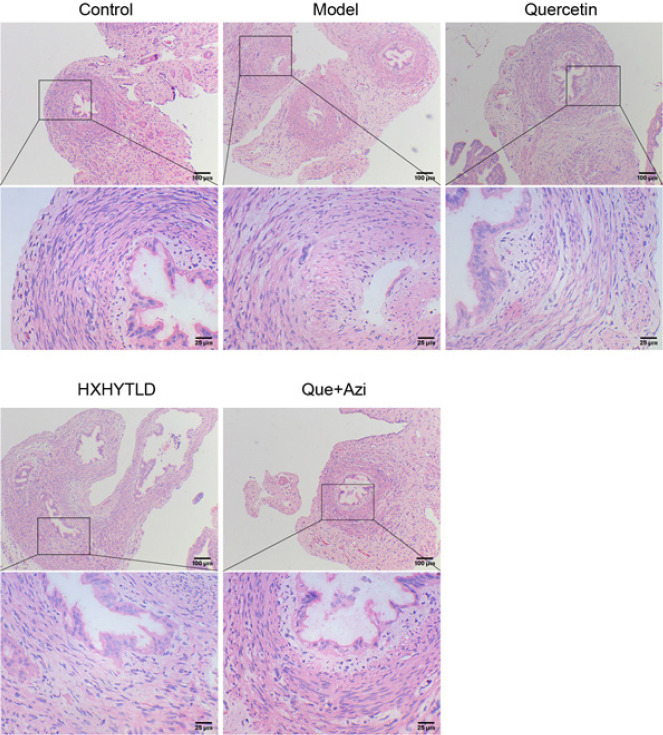
Combination therapy of quercetin and azithromycin counteracted the histomorphological change of hydrosalpinx

To further characterize the function of the quercetin and azithromycin therapy in treating TFI rats, we first examined the pathological changes in the fallopian tube. After randomly sacrificing SD rats in the Model group, we observed that the Model showed a swelling fallopian tube with hydrosalpinx (Figure 3), indicating hydrosalpinx formation due to inflammation. Using the ​Hematoxylin and Eosin (H&E) stain, we proceeded to observe the histomorphological changes in the tissues of fallopian tubes in each experimental group (Figure 4). In the model tissue, hydrosalpinx was developed. Additionally, it was observed that the intraluminal epithelium mainly contained flat epithelial cells, suggesting a morphological change, and shedding of the epithelial cells of the lamina propria. There existed lumen stenosis in the model tissue with severe epithelial damage (Figures 3 and 4). However, both quercetin and Huoxuehuayu Tongluo Decoction exhibited the ability to maintain integrity, which showed the phenomenon of lumen stenosis had also been largely reduced (Figures 3 and 4). Interestingly, using quercetin and Azithromycin, the tissues had been maximally protected from developing hydrosalpinx (Figure 3). The histomorphology of the fallopian tube tissue was maintained without significant damage because no lumen stenosis was observed in the tissues from the corresponding group (Figure 4). Thus, these findings suggest that quercetin and Huoxuehuayu Tongluo Decoction, especially the quercetin and azithromycin therapy, effectively counteract the development of hydrosalpinx and maintain the integral structure of the fallopian tube tissue in TFI rats. 

***Quercetin and azithromycin combination therapy prevents fallopian tube tissue damage***

**Figure 5:**
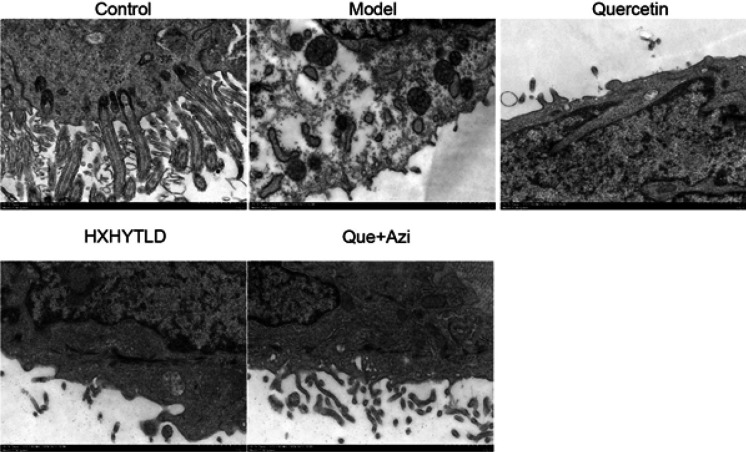
Observation of fallopian tube tissue structures with the treatment of the quercetin and azithromycin combination therapy using EM

To solidify the HE staining findings, we used electron microscopy to observe further the morphological changes in fallopian tube tissues in the Model and the treatment groups (Figure 5). In the normal tissue, epithelial cells featured long cilia and dense microvilli (Control), while abnormal epithelial cells of fallopian tube tissues barely had such structures (Model)(Figure 5). There were exposed and necrotic epithelial areas in the abnormal fallopian tube tissue, and the eroded epithelial surface showed stomata exuding spheroids (Figure 5). Quercetin and azithromycin combination therapy notably prevented the tissue from developing hydrosalpinx, which showed the entire tissue structure was well maintained, with long cilia and dense microvilli preserved (Figure 5). Although a large portion of the tissue structure remained integral, long cilia and dense microvilli were much less than in the Control group and the Quercetin and Azithromycin combination therapy group (Figure 5). Based on these microscopy results, it is convincing that quercetin and azithromycin combination therapy could protect fallopian tube tissues from being damaged by inflammation. 

***Quercetin and azithromycin combination therapy inhibits the release of various pro-inflammatory cytokines***

**Figure 6:**
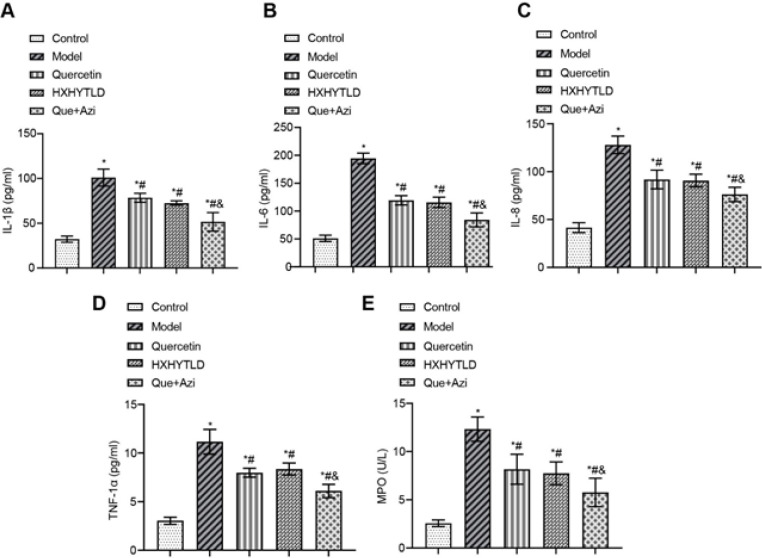
Levels of the release of multiple pro-inflammatory cytokines were reduced in the presence of the quercetin and azithromycin combination therapy of tubal factor infertility (TFI) rats

The therapy of quercetin and Huoxuehuayu Tongluo Decoction, especially the quercetin and azithromycin combination therapy, demonstrated strong inhibitory effects on the development of inflammation in rat fallopian tubes. As a result, we further advanced our research by measuring the release of pro-inflammatory cytokines by ELISA assay. All these pro-inflammatory cytokines usually trigger inflammation in fallopian tubes, including IL-1β (Figure 6A), 1L-6 (Figure 6B), IL-8 (Figure 6C), TNF-α (Figure 6D), and MPO (Figure 6E). However, in the Model group, all cytokines reached significantly high levels, which were 2–4 folds of Control, indicating that the inflammation of the fallopian tube was highly correlated with the release of cytokines (Figure 6). The use of individual treatments was able to significantly mitigate the release of these cytokines, which lowered the release levels of cytokines by 25% ~ 35% compared with the Model (Figure 6C). More strikingly, the combination therapy showed the most potency in reducing cytokine release, which led to a remarkable decrease of about 45% (Figure 6C). Therefore, it was convincing that the quercetin and azithromycin combination therapy could effectively reduce pro-inflammatory cytokine release to further inhibit inflammation in TFI rat fallopian tubes.

***Quercetin and azithromycin combination therapy inhibits inflammatory signaling pathways***

**Figure 7:**
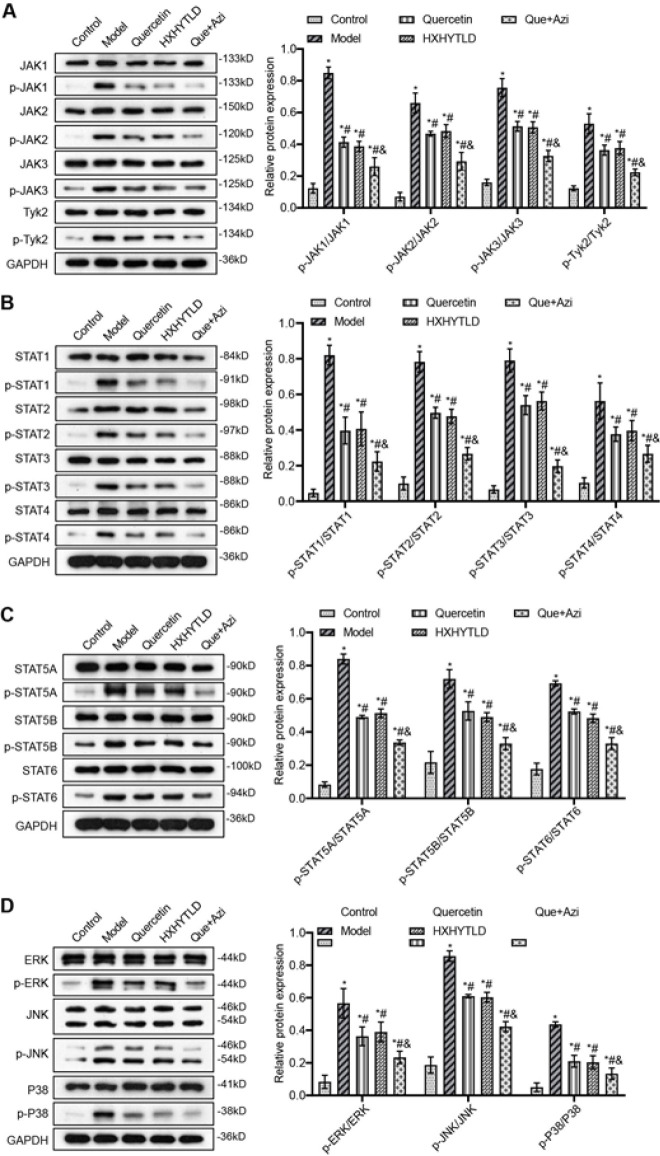
Quercetin and azithromycin combination therapy significantly inhibits the phosphorylation of effector proteins in important signaling pathways

The characterization of the inhibitory effects of quercetin, Huoxuehuayu Tongluo Decoction, and quercetin and azithromycin combination therapy on the release of pro-inflammatory cytokines inspired us to investigate the transduction pathways involved in the entire process and the regulatory mechanism behind it (Figure 7). We first targeted the JAK-STAT signaling pathway, which is tightly associated with regulating several pro-inflammatory cytokines, including IL-1β, IL-6, and TNF-α. Compared with Control, the phosphorylation levels were more than 4 folds higher in the Model. The phosphorylation levels of JAK proteins and Tyk2 dropped significantly due to different therapies (Figure 7A). In terms of potency, either quercetin or Huoxuehuayu Tongluo Decoction generated an equivalent inhibition, while the quercetin and azithromycin combination therapy showed the highest activity (Figure 7A). Similarly, the phosphorylation of downstream STAT proteins was significantly inhibited after treatment (Figures 7B and 7C). Especially, the phosphorylation level of STATs was down-regulated considerably by the quercetin and azithromycin combination therapy (Figures 7B and 7C). In addition to the JAK-STAT pathway, we also determined critical proteins related to the MAPK signaling pathway, including ERK, JNK, and p38 (Figure 7D). Consistent with the results of the JAK-STAT phosphorylation, the phosphorylation of these proteins was inhibited, in which quercetin and azithromycin combination therapy elicited the highest inhibition (Figure 7D). As a result, all the therapies used for inflammation treatment, especially the quercetin and azithromycin combination therapy, enable potent inhibition of different pro-inflammatory signalings to mitigate the inflammation of fallopian tubes further.

***Quercetin and azithromycin combination therapy inhibits the nuclear translocation of NF-*** ***κB*** ***p65***

**Figure 8:**
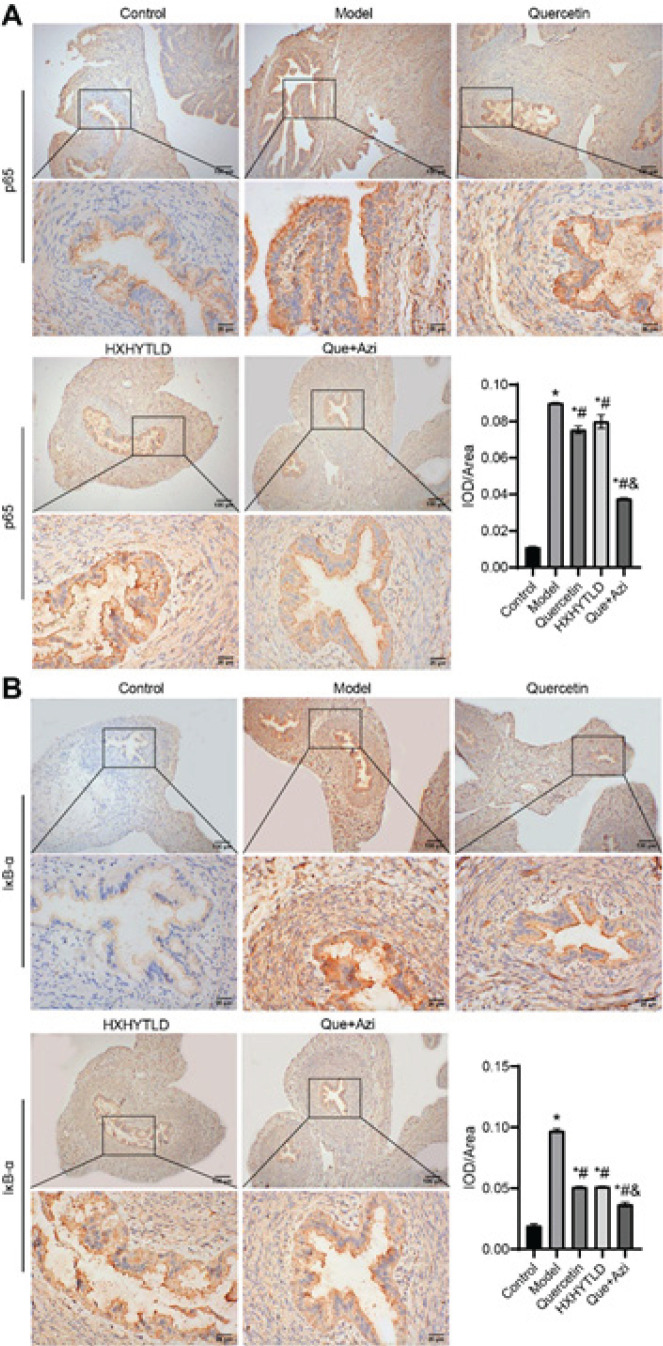
Quercetin and azithromycin combination therapy inhibited the nuclear translocation of NF-κB p65

To elucidate the relationship between Huoxuehuayu Tongluo Decoction and NF-κB signaling, we performed immunohistochemistry (IHC) to identify the enrichment of p65 in the nucleus (Figure 8A). In the ciliated columnar epithelium of lamina propria, no obvious nuclear translocation was discovered in Control (Figure 8A). In comparison, we observed tremendous enrichment of NF-κB p65 in the Model group (Figure 8A). The use of quercetin or Huoxuehuayu Tongluo Decoction decreased the translocation, but the quercetin and azithromycin combination therapy more significantly prevented this process, indicating our therapy’s capability of inhibiting the NF-κB pathway and relevant downstream transcription activity (Figure 8A). In the meantime, we also determined the level of IκBα in different groups to substantiate the findings above (Figure 8B). In contrast to Control, the Model demonstrated the highest IκBα distribution (Figure 8B). With the use of the therapies, it is noted that the detectable level of IκBα had been largely recovered, indicating that the translocation of p65 was reduced when using the medicines (Figure 8B). Likewise, the quercetin and azithromycin combination therapy rendered the best results (Figure 8B). Taken together, it was suggested that the quercetin and azithromycin combination therapy had demonstrated the efficacy of inhibiting the NF-κB pathway to suppress the downstream transcription and translation of inflammation-related proteins.

**Table 1:** Basic information of primary antibodies in Western blot

Name	**Catalog No.**	**Animal**	**Dilution ratio**	**Supplier**	**C** **ountry**
Anti-STAT1	10144-2-AP	Rabbit	1:1000	proteintech	USA
Anti-p-STAT1	ab109461	Rabbit	1:4000	Abcam	UK
Anti-STAT2	ab32367	Rabbit	1:6000	Abcam	UK
Anti-p-STAT2	#88410	Rabbit	1:1000	Cell Signaling Technology	USA
Anti-STAT3	10253-2-AP	Rabbit	1:3000	proteintech	USA
Anti-p-STAT3	ab76315	Rabbit	1:4000	Abcam	UK
Anti-STAT4	13028-1-AP	Rabbit	1:1000	proteintech	USA
Anti-p-STAT4	ab194732	Rabbit	1:2000	Abcam	UK
Anti-STAT5A	13179-1-AP	Rabbit	1:1500	proteintech	USA
Anti-p-STAT5A	ab30648	Rabbit	1:1000	Abcam	UK
Anti-STAT5B	12071-1-AP	Rabbit	1:1000	proteintech	USA
Anti-p-STAT5B	ab52211	Rabbit	1:1000	Abcam	UK
Anti-STAT6	51073-1-AP	Rabbit	1:2000	proteintech	USA
Anti-p-STAT6	ab263947	Rabbit	1:1000	Abcam	UK
Anti-GAPDH	10494-1-AP	Rabbit	1:4000	proteintech	USA
Anti-JAK1	66466-1-Ig	Mouse	1:2000	proteintech	USA
Anti-p-JAK1	ab138005	Rabbit	1:3000	Abcam	UK
Anti-JAK2	17670-1-AP	Rabbit	1:1000	proteintech	USA
Anti-p-JAK2	ab32101	Rabbit	1:3000	Abcam	UK
Anti-JAK3	ab203611	Rabbit	1:750	Abcam	UK
Anti-p-JAK3	bs-20168R	Rabbit	1:1000	Bioss	China
Anti-Tyk2	bs-6662R	Rabbit	1:1000	Bioss	China
Anti-p-Tyk2	bs-3437R	Rabbit	1:1000	Bioss	China
Anti-ERK	11257-1-AP	Rabbit	1:1000	proteintech	USA
Anti-p-ERK	ab50011	Mouse	1:4000	Abcam	UK
Anti-JNK	ab179461	Rabbit	1:1000	Abcam	UK
Anti-p-JNK	ab124956	Rabbit	1:4000	Abcam	UK
Anti-P38	14064-1-AP	Rabbit	1:1500	proteintech	USA
Anti-p-P38	ab4822	Rabbit	1:1000	Abcam	UK
HRP goat anti-mouse IgG	SA00001-1	Goat	1:5000	proteintech	USA
HRP goat anti-rabbit IgG	SA00001-2	Goat	1:6000	proteintech	USA

**Table 2:** Key component information of Huoxuehuayu Tongluo Decoction in tubal factor infertility (TFI) disease

MOL ID	Name	Average shortest path length	Betweenness centrality	Closeness centrality	Degree
MOL000098	Quercetin	2.03876	0.094378	0.490494	55
MOL000006	Luteolin	2.263566	0.025922	0.441781	27
MOL000422	Kaempferol	2.209302	0.032628	0.452632	25
MOL013179	Fisetin	2.348837	0.016235	0.425743	19

(A) Venn diagram showed the targets between Huoxuehuayu Tongluo Decoction and TFI. (B) PPI network analysis showed the key gene targets. (C) Component-disease target network between Huoxuehuayu Tongluo Decoction drug components and TFI disease targets

(A) Degree of the target gene in TFI diseases. (B) Molecular docking between quercetin and IL-6. (C) Analysis of KEGG function prediction

**Table 3:** Pregnancy rates and average litter sizes in different groups

Group	Pregnancy rate (%)	Average litter size (Mean ± SD)
Control	100	12.60 ± 1.51
Model	40	7.75 ± 0.96
Quercetin	70	9.57 ± 0.98
HXHYTLD	70	9.71 ± 1.11
Que+Azi	80	10.75 ± 1.49

HXHYTLD represented Huoxuehuayu Tongluo Decoction

H&E staining of the structure of rat fallopian tissues. Scale bar: 100 μm for the upper panel and 25 μm for the lower panel. HXHYTLD represented Huoxuehuayu Tongluo Decoction

Histomorphological changes in the structure of rat fallopian tissues of each experimental group under TEM. Scale bar: 1 μm. HXHYTLD represented Huoxuehuayu Tongluo Decoction

The cellular levels of IL-1β (A), IL-6 (B), IL-8 (C), TNF-α (D), and MPO (E) were determined by ELISA. *P<0.05, compared with the Control group; ^#^*P<*0.05, compared with the Model group; ^&^*P<*0.05 compared with the HXHYTLD or quercetin groups. HXHYTLD represented Huoxuehuayu Tongluo Decoction

(A) Western blot of the phosphorylation of JAK1-3 and Tyk2. (B-C) Western blot of the phosphorylation of STAT1-6. (D) Western blot of the phosphorylation of ERK, JNK, and p38. *P<0.05, compared with Control; ^#^*P<*0.05, compared with Model; ^&^*P<*0.05 compared with HXHYTLD or quercetin groups. HXHYTLD represented Huoxuehuayu Tongluo Decoction

(A) IHC of NF-κB p65 level in rat fallopian tissues. (B) IHC of IκBα level in rat fallopian tissues. HXHYTLD represented Huoxuehuayu Tongluo Decoction

## Discussion

Conventionally, the treatment of PID-induced TFI would rely on various combinations of antibiotics based on the instruction of the Centers for Disease Control and Prevention (https://www.cdc.gov/std/tg2015/pid.htm). However, the listed antibiotics have different side effects that induce discomfort and even severe pain. For example, cefoxitin may cause swelling, redness, pain, or even loss of appetite, nausea, vomiting, diarrhea, or headache (39). Instead of targeting specific disease-causing agents, the treatment with TCM establishes the concept of assisting the body to maintain health, which is better in curing the root of the problem (40, 41). Our data captured drastic decreases in the release of critical cytokines that induce inflammation in fallopian tubes, indicating that Huoxuehuayu Tongluo Decoction or the quercetin of Huoxuehuayu Tongluo Decoction and azithromycin combination therapy had the inhibitory function of reducing the levels of these factors. All the studies proved the combination therapy of quercetin and azithromycin dredged collateral, which improved the pregnancy rates in the modeled rats with hydrosalpinx in fallopian tubes, which could be a new therapy in TFI.

Huoxuehuayu Tongluo Decoction proved effective in attenuating the symptoms of inflammation (18, 42). The formation of inflammation is a complicated process involving a wide spectrum of pro-inflammatory cytokines (43). The release of these cytokines is tightly associated with various signaling pathways (44, 45). Cai *et al*. (18) reported the use of Huoxuehuayu Decoction for nonalcoholic fatty liver disease (NAFLD) in patients, a disorder characterized by liver inflammation (46). Our study showed that quercetin was the key component with the highest degree in Huoxuehuayu Tongluo Decoction, which was bound with IL-6, suggesting a potential mechanism for its role in TFI . It was determined that the therapeutic effectiveness of Huoxuehuayu Tongluo Decoction on inflammatory disease largely depends on its efficacy of inhibiting pro-inflammatory signaling pathways by down-regulating the phosphorylation of critical effector proteins. 

The cytokine IL-6 is an important mediator of pro - and anti-inflammatory processes (47). Dys-regulated IL-6-induced intracellular JAK/STAT signaling is associated with severe inflammatory disease (48). As a classic and one of the most characterized cellular signaling pathways, JAK-STAT is a vital participant in regulating inflammatory reactions (49). IL-6-treated macrophages induced phospho-STAT3 nuclear translocation and transcriptional activity, and increased the cellular abundance of phospho-P38 MAPK (50). MAPK signaling regulates multiple important cellular processes, including proliferation, differentiation, migration, apoptosis, and inflammation, rendering it a competent target for treating inflammatory disease (51). Our research observed declining phosphorylation of JNK, ERK, and p38 when Huoxuehuayu Tongluo Decoction was applied, possibly due to the combination of quercetin and IL-6, which provided new methods for TFI treatment. 

However, the specific regulatory mechanism of quercetin with IL-6 in TFI still needs further study, which was a limitation in this study. IL-6 transduction signaling mechanisms activate various pathological pathways, such as JAK/STAT3, Ras/MAPK, and PI3K-PKB/Akt, and regulate CD4+T cell and VEGF levels, leading to cancer, inflammatory diseases, and neurological diseases (52). IL-6 significantly down-regulates many transporters (ABC and SLC) in the liver through co-activation of STAT3/NF-κB(53). Quercetin exposure reduced IL-6 activation of GP130, JAK1, and STAT3, as well as significantly reducing IL-6-induced proliferation and migration characteristics of glioblastoma cells(54, 55). The involvement of IL-6 in the pathophysiology of these complex diseases makes it an important target for the treatment of these diseases. Therefore, it was necessary to further explore the role of quercetin or quercetin and azithromycin in IL-6 transduction mechanisms for the treatment of TFI. 

To sum up, all these findings have reinforced the notion that quercetin and azithromycin were potent in treating the hydrosalpinx of fallopian tubes. Our current study revealed the key role of the therapy in promoting the pregnancy rate of rats that suffer from TFI, which represented the first attempt to utilize TCM for treating hydrosalpinx of fallopian tubes. The unveiling of the inhibitory function of the quercetin and azithromycin combination therapy on multiple critical inflammation-associated signaling pathways provided insights and prospects for treating human TFI. 

## Conclusion

Quercetin and azithromycin combination therapy inhibited inflammation and phosphorylation of JAK/STAT and MAPK pathways to improve TFI inflammation and pregnancy function. This study focused on the quercetin and azithromycin combination therapy and provided insights and prospects for treating human TFI.

## Data Availability Statement

The data that support the findings of this study are available from the corresponding author upon reasonable request.

## Funding Sources

This work was supported by the National Natural Science Foundation of China (81804140), the Natural Science Foundation of Hunan Province (2018JJ3407), the Project of Hunan Province “Shennong Talents” for Young Shennong Scholars, the Project of Hunan Province “Hunan Young Talents”, the Research Project on Chinese Medicine of Hunan Province (A2023052), and the Scientific Research Fund of Hunan Provincial Health Department (20232627). 

## Authors’ Contributions

L S contributed to data collection, data analysis, and manuscript writing; N W, Y Y, and Y T helped with project development, data analysis, manuscript writing, and manuscript editing; Q W, L-Lei, and M W performed data analysis and manuscript editing; L-Liu contributed to project development, data analysis, and manuscript editing.

## Conflicts of Interest

The authors declare that there are no conflicts of interest in this work.
